# The Graphene Oxide/Gold Nanoparticles Hybrid Layers for Hydrogen Peroxide Sensing—Effect of the Nanoparticles Shape and Importance of the Graphene Oxide Defects for the Sensitivity

**DOI:** 10.3390/molecules30030533

**Published:** 2025-01-24

**Authors:** Krystian Pupel, Kacper Jędrzejewski, Sylwia Zoladek, Marcin Palys, Barbara Palys

**Affiliations:** Faculty of Chemistry, University of Warsaw, Pasteur Str. 1, 02-093 Warsaw, Poland; k.pupel@uw.edu.pl (K.P.); kk.jedrzejewski@uw.edu.pl (K.J.); szoladek@chem.uw.edu.pl (S.Z.); mpalys@chem.uw.edu.pl (M.P.)

**Keywords:** hydrogen peroxide sensing, graphene, gold nanoparticles, Raman intensities, non-enzymatic sensor, reactive oxygen species detection

## Abstract

Graphene oxide (GO) and reduced graphene oxides (RGOs) show intrinsic electrocatalytic activity towards the electrocatalytic reduction of H_2_O_2_. Combining these materials with gold nanoparticles results in highly sensitive electrodes, with sensitivity in the nanomolar range because the electrocatalytic properties of GO and nanoparticles are synergistically enhanced. Understanding the factors influencing such synergy is crucial to designing novel catalytically active materials. In this contribution, we study gold nanostructures having shapes of nanospheres (AuNSs), nanourchins (AuNUs), and nanobowls (AuNBs) combined with GO or electrochemically reduced graphene oxide (ERGO). We investigate the amperometric responses of the hybrid layers to H_2_O_2_. The AuNUs show the highest sensitivity compared to AuNBs and AuNSs. All materials are characterized by electron microscopy and Raman spectroscopy. Raman spectra are deconvoluted by fitting them with five components in the 1000–1800 cm^−1^ range (D*, D, D”, G, and D′). The interaction between nanoparticles and GO is visualized by the relative intensities of Raman bands (ID/IG) and other parameters in the Raman spectra, like various D”, D* band positions and intensities. The ID/IG parameter is linearly correlated with the sensitivity (R^2^ = 0.97), suggesting that defects in the graphene structure are significant factors influencing the electrocatalytic H_2_O_2_ reduction.

## 1. Introduction

Starting from food safety monitoring and product quality control [1], H_2_O_2_ sensors have become essential tools in diversified research areas down to detailed studies in cellular signaling and oxidative stress within biological contexts [2]. H_2_O_2_, an active endogenous species, is an important participant in various biochemical processes in living organisms [3]. However, due to its bifunctional nature, it is not only a critical signaling molecule but also a potentially harmful one. While controlled, low concentrations of H_2_O_2_ participate in cellular communication and fulfill various regulatory functions; high concentrations may initiate oxidative stress, damaging DNA, proteins, and lipids, or leading to cell death [4]. This duality demands the precise monitoring and accurate detection of H_2_O_2_ concentrations within biological, industrial, and environmental contexts.

Hydrogen peroxide, H_2_O_2_, is an important by-product of enzymatic reactions, especially glucose oxidation [5]. The precise sensing of H_2_O_2_ is very important in medical diagnosis, especially in managing diabetes, wherein glucose sensors depend upon the correct measurement of this very molecule [5]. Besides medical applications, hydrogen peroxide is an essential indicator of oxidative stability in the pharmaceutical industry and in environmental studies, showing the presence of pollutants in the water systems [6,7]. It can indicate, for example, the contamination through industrial effluents that eventually affects ecosystems and thus requires reliable detection methods. For studies of biological samples, the sensors should operate at a neutral or slightly acidic pH [5,8].

Among various detection methodologies, electrochemical sensors are one of the primary approaches to H_2_O_2_ detection thanks to convenience in construction, low operation cost, and fast response [9]. Various types of electrodes with catalytic properties toward reducing H_2_O_2_ were used in such sensors [9]. Gold nanoparticles (AuNPs) have gradually become among the best candidates because of their excellent chemical stability, high conductivity, and good catalytic activity [10,11,12,13]. The advantage of AuNPs in enhancing the sensitivity of sensors is that they allow the development miniaturized sensors suitable for portable devices [14].

Recent research has also focused on how nanoparticles’ morphology influences the sensors’ performance. For instance, Won et al. demonstrated that gold nanorods (AuNRs) are more sensitive to H_2_O_2_ than gold nanospheres (AuNSs) [12]. Their enhanced performance may be attributed to AuNRs’ higher conductivity, and the larger surface area of the nanoparticles allows for the more active interaction of the nanoparticles with H_2_O_2_ molecules [15]. The results highlight that nanoparticle morphology is crucial to sensor performance [16]. According to our knowledge, the effect of nanoparticle shapes has not been systematically studied to date.

In parallel, graphene-related materials have recently gained high interest due to their unique properties, such as excellent electrical conductivity, high specific surface area, and the abundance of functional groups, making graphene-based materials especially suitable for electrode modifications in biosensor applications [17]. Nitrogen-doped graphene and chemically reduced graphene oxide conjugated with AuNPs showed a peroxidase-like activity comparable to horseradish peroxidase, well known as a standard enzyme in biosensing [18].

Graphene oxide (GO) and electrochemically reduced graphene oxide (ERGO) show intrinsic electrocatalytic activity towards the H_2_O_2_ reduction. However, the individual performance of both these electrodes was not satisfactory compared with that of gold nanoparticles [14,19,20]. Combining graphene-based materials with gold nanoparticles resulted in a synergy significantly improving sensor sensitivity [21]. The effect is due to the complementary function played by these materials: while gold nanoparticles provide active catalytic sites, graphene enhances electron transfer kinetics and offers a supporting matrix for nanoparticle dispersion [22]. It is known that the synergy effect depends on the reduction procedure of GO and on the pretreatment of the electrocatalytic layer. Still, the mechanism of the synergic effect remains unclear.

The novel approaches for H_2_O_2_ detection include the use of AuNPs stabilized with peroxometalate anions in conjunction with electrochemically reduced graphene oxide, which proved quite effective and provided very sensitive detection, hence measuring even nanomolar concentration levels of H_2_O_2_ [23,24]. These ultra-sensitive detection systems hold immense possibilities for application areas where detecting trace amounts of H_2_O_2_ becomes imperative, including medical diagnosis, environmental testing, and pharmaceutical analysis.

**Scheme 1:**
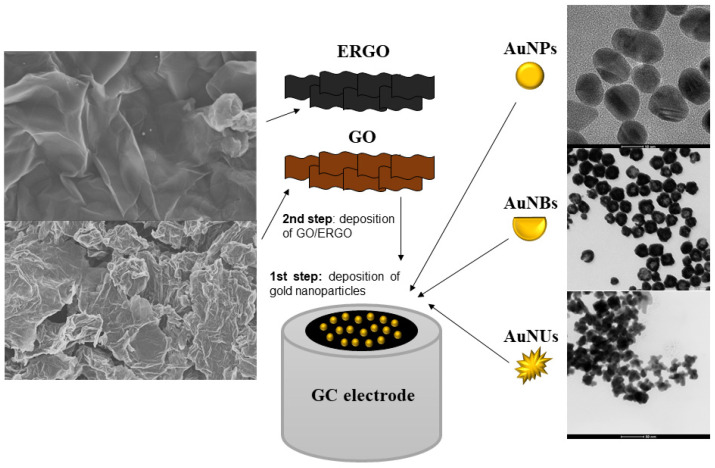
Overview of the studied systems.

This study examines the electrocatalytic efficiency of three different shapes of gold nanoparticles, including gold nanospheres (AuNSs), gold nanobowls (AuNBs), and gold nanourchins (AuNUs), having similar sizes. We describe how morphology can enable synergistic electrocatalysis toward the reduction of H_2_O_2_ and analyzes different shapes individually and as composites with GO or ERGO. The experiments are carried out at pH = 6.5, which is suitable for H_2_O_2_ determination in a wide range of biological systems [10]. The overview of the studied systems is shown in Scheme 1. The relationship between sensor sensitivity and variables obtained with Raman spectroscopy is also studied in this work, in an attempt to extend the understanding of contribution of those synergies.

## 2. Results and Discussion

### 2.1. Characterization and Cyclic Voltammetry Responses of Studied Electrodes

**Figure 1:**
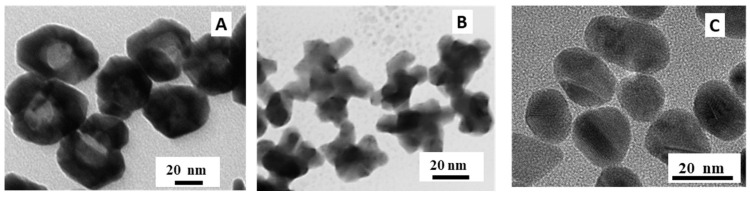
TEM pictures of the studied gold nanoparticles: AuNBs (**A**); AuNUs (**B**), and AuNSs (**C**).

To study the effect of the shape of gold nanostructures on the electrocatalytic properties, we chose the AuNBs, AuNUs, and AuNSs with similar dimensions. Their shapes are illustrated in Figure 1. The AuNUs have the most developed surface area compared to AuNBs and AuNSs. All nanoparticles were chemically synthesized (see Section 3).

**Figure 2:**
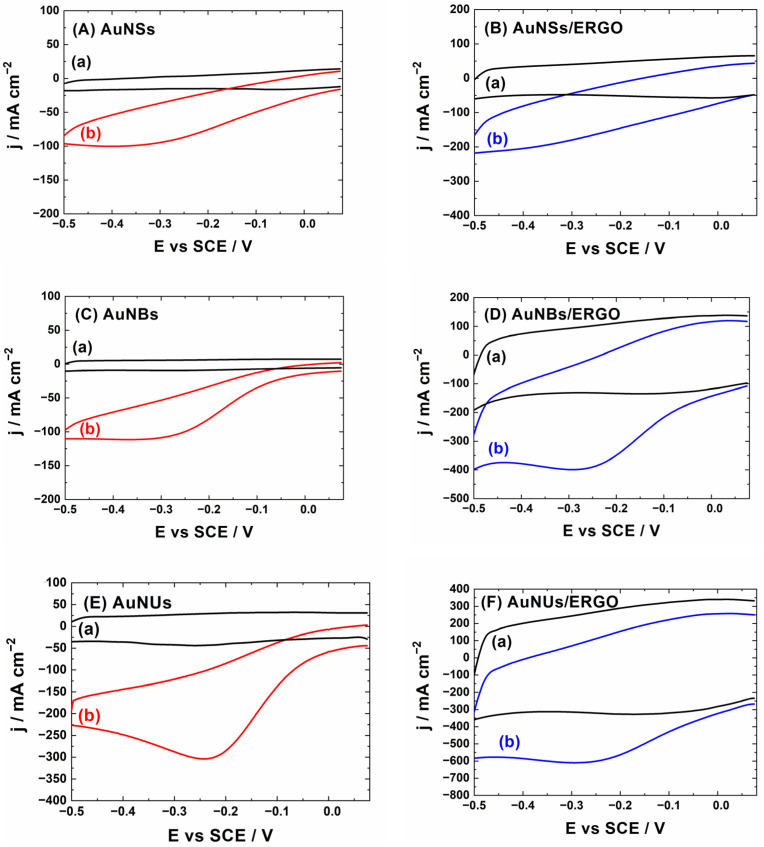
Cyclic voltammetry responses of (**A**) AuNSs; (**B**) AuNSs/ERGO; (**C**) AuNBs; (**D**) AuNBs/ERGO; (**E**) AuNUs; (**F**) AuNUs/ERGO layers on glassy carbon electrodes in de-aerated phosphate buffer at pH = 6.5 (a) without H_2_O_2_ and (b) with 2 mM of H_2_O_2_. Scan rate 10 mV/s. The current density is calculated using the geometric surface area of electrodes.

Figure 2 shows the cyclic voltammograms of the studied layers without H_2_O_2_ in the supporting electrolyte (black curves) and in the supporting electrolyte containing 2 mM of H_2_O_2_. Without H_2_O_2_ in the solution, all cyclic voltammograms show only a capacitive current in the studied potential range. Such an observation is consistent with the findings of other authors [25] and our previous results [23]. Comparing the cyclic voltammograms of bare AuNSs, AuNBs, and AuNUs layers (Figure 2A,C,E respectively) with the voltammograms of the nanoparticles covered with the ERGO layers (Figure 2B,D,F respectively), it can be noticed that ERGO causes a significant increase in the capacitive current. It is generally accepted that GO or reduced GO layers are characterized by high capacitive currents due to abundant oxygen surface groups [26]. The ERGO layers have a rather low oxygen content, but they contain multiple defects, which influence the adsorption of ions, and leads to the increase in the capacitive current. The layers of the gold nanoparticles covered by ERGO show a significantly higher capacitive current, probably due to the abundant ion adsorption on the ERGO surface.

The increase in the current intensity in the presence of H_2_O_2_ is visible for all studied electrodes. The onset of the current of H_2_O_2_ reduction, at ca. 0.0 V vs. SCE, is similar for all electrodes, while current intensities significantly differ. If layers of AuNSs (Figure 2A), AuNBs (Figure 2B), and AuNUs (Figure 2C) without ERGO are compared, AuNUs show the highest reduction current. The maximal current value for AuNUs exceeds −300 µA·cm^−2^, while for AuNSs, it is ca. −100 µA·cm^−2^. The enhanced activity observed for AuNUs can be explained by their shape (high surface area and sharp edges). Some sharp edges are also characteristic for AuNBs, which show higher currents comparing to AuNSs but lower than AuNUs. We suppose that the presence of edges is important, because they expose under-coordinated gold atoms, a prerequisite for the catalytic activity of gold nanoparticles [27]. Cyclic voltammetry curves for AuNSs and AuNBs show a wave-like response. In contrast, the curve for AuNUs is peak-shaped, suggesting that the electrochemical reaction is limited by the diffusion of a substrate. This further indicates that the reaction of the H_2_O_2_ reduction on AuNUs is faster compared to AuNBs and AuNSs, where it is limited kinetically. The layers covered by the ERGO layer (Figure 2B,D,F) resemble those of the corresponding bare nanoparticles (Figure 2A,C,E), but their shape is distorted by the high capacitive current. This observation suggests that the ERGO layer influences the adsorption of species contributing to the electrode capacity, but the gold nanoparticles determine the kinetics of the H_2_O_2_ electroreduction.

### 2.2. Amperometric Responses to H_2_O_2_

**Figure 3:**
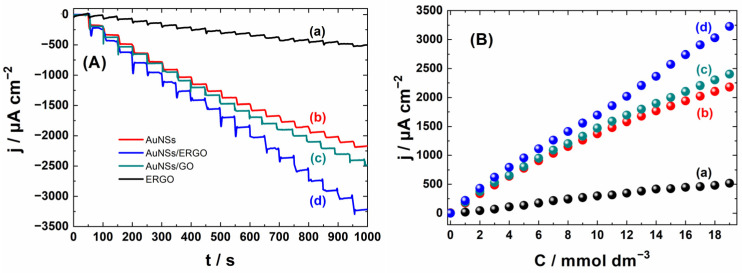
Amperometric responses (**A**) of ERGO (a); AuNUs (b); AuNUs/GO (c); and AuNUs/ERGO (d) with the corresponding calibration plots (**B**). The current density is calculated using the geometric surface area of electrodes.

Following the cyclic voltammetry results, we chose the −0.2 V vs. SCE potential value to study amperometric response to H_2_O_2_ as the current is high at this potential value. The typical amperometric results for layers containing AuNUs are shown in Figure 3, together with the corresponding calibration plots. The results for the bare ERGO layer are shown for comparison (plot a in Figure 3). The sensitivity of ERGO is visible, though it is significantly smaller than that of the electrodes with gold nanostructures. The sensitivity of GO is even lower than that of ERGO, which can be connected to the poor conductivity of GO. The worse conductivity of GO than of the reduced GOs is generally acknowledged [28]. The weak response of GO to H_2_O_2_ can be also related to other properties affecting the electrocatalytic reaction.

Despite the low conductivity of GO, depositing the GO layer on AuNUs enhances the amperometric response of AuNUs (curves b and c in Figure 3), suggesting synergy in the electrocatalytic activity. The beneficial effect of GO indicates that this material improves the adsorption and diffusion of H_2_O_2_ in the electrocatalytic layer. This enhancement of the amperometric response is much more significant in the case of ERGO: it probably improves the adsorption and diffusion of H_2_O_2_ like GO, but it is more conductive than GO. The currents recorded for the AuNUs/ERGO layer are typically higher than the sum of the current for ERGO and AuNUs at the same concentration of H_2_O_2_, indicating the synergy effect between ERGO and AuNUs.

**Figure 4:**
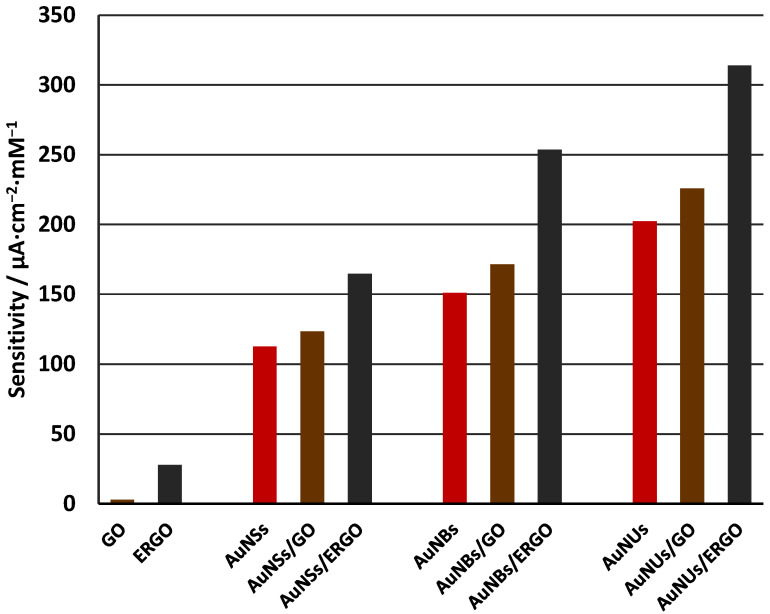
The overview of the sensitivities of studied electrodes.

Similar dependencies are observed for AuNSs and AuNBs. The slopes of calibration plots for all studied electrodes are collected in Table 1 to facilitate the comparison. The overview of the sensitivities is shown in the column plot in Figure 4.

Sensitivity clearly depends on the shape of the gold nanoparticles in the order: AuNSs < AuNBs < AuNUs. We attribute this effect to the surface area of the studied nanoparticles. The AuNUs have a branched morphology, as illustrated in Figure 1. The surface area of such nanoparticles is thus very high. On the contrary, the surface area of spherical nanoparticles is the smallest. The order of sensitivities thus follows the order of the surface area of the nanoparticles, suggesting that the surface is the most important factor responsible for the differences in the electrocatalytic properties of AuNSs, AuNBs, and AuNUs. Besides the surface area, the presence of sharp edges in AuNUs and AuNBs also contributes to the enhanced activity of these nanoparticles due to the high content of the under-coordinated gold atoms [27].

In all cases, covering the gold nanoparticles with the layer of GO enhances the sensitivity despite its relatively poor conductivity. If the ERGO layer is used, a further increase in the sensitivity is observed. We suppose that both GO and ERGO contribute to the mechanism of the H_2_O_2_ reduction by facilitating the adsorption of H_2_O_2_. The mechanism of the electrocatalytic reaction involves following steps, as suggested in the references [29,30,31]:

H_2_O_2_ + e^−^ → OH_ad_ + OH^−^ (1)

OH_ad_ + e^−^ → OH^−^ (2)

2OH^−^ + 2H^+^ → 2H_2_O (3)

Reaction (1) is the rate limiting step. We suppose that depositing of GO or ERGO on the layers of gold nanoparticles facilitates this reaction, resulting in higher reduction currents. This is the synergetic electrocatalytic effects of the GO (or ERGO) layer and the gold nanoparticles. It is noticeable that for all AuNSs, AuNBs, and AuNUs, covering the nanoparticles with the ERGO layer results in very high signals, higher than the sum of the sensitivities of the bare nanoparticle layer plus the sensitivity of the bare ERGO layer. This indicates the synergy between the nanoparticles and ERGO. In the case of AuNUs, the sensitivity increase due to the presence of ERGO is very high, being more than 100 µA·cm^−2^·mM^−1^. The synergy between AuNUs and ERGO is thus very high.

To investigate the effect of possible differences of electrodes’ real surface areas on the sensitivity, we measured cyclic voltammograms in the solution containing the 0.01 M K_2_[Fe(CN)_6_]. The real surface *A* was estimated from Randless–Sevcik equation [32] in the form *Ip = 2.69·10^5^D^1/2^n^3/2^CAv^1/2^*, where *Ip* is the peak current of the K_2_[Fe(CN)_6_] oxidation, *D* is the diffusion coefficient of [Fe(CN)_6_]^4−^ ions (equal to 6.05·10^−6^ cm^2^ s^−1^), *n* is the number of electrons exchanged in the reaction (*n* = 1 for the concerned reaction), *C* is the concentration of K_2_[Fe(CN)_6_], and *v* is the scan rate in V/s. The resulting surface areas are collected in Table 2. The comparison of Table 1 and Table 2 shows that sensitivity to H_2_O_2_ is not correlated with the real electrode surface area. The electrodes having nearly identical surface areas, for example, ERGO, AuNBs/ERGO, and AuNUs/GO, have very different sensitivities to H_2_O_2_. This observation suggests that other properties like the presence of defects or active sites on gold nanoparticles (the under-coordinated gold atoms) are more important for the electrocatalytic activity.

### 2.3. Correlation Between the Raman Spectra of the AuNPs—GO or ERGO Layers and Their Sensitivity to H_2_O_2_

**Figure 5:**
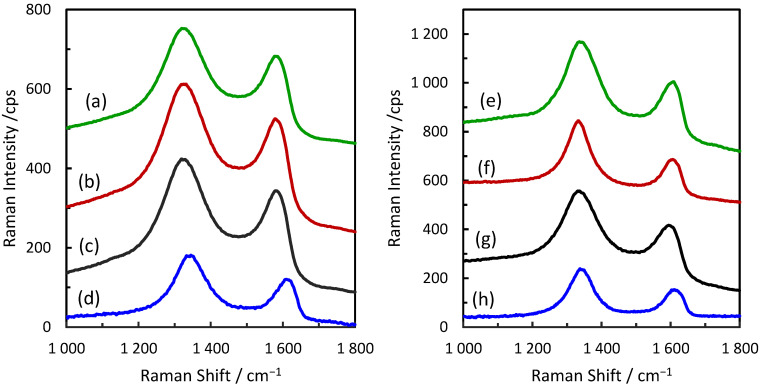
Raman spectra of the studied electrodes: (a) AuNUs/GO; (b) AuNBs/GO; (c) AuNSs/GO; (d) GO; (e) AuNUs/ERGO; (f) AuNBs/ERGO; (g) AuNSs/ERGO; and (h) ERGO.

**Figure 6:**
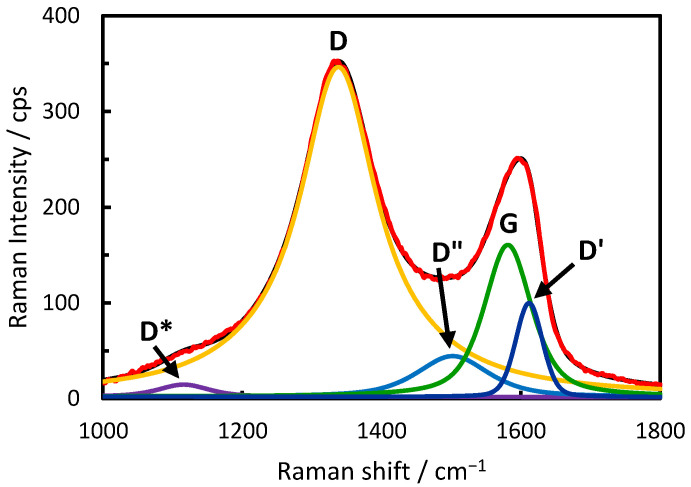
Fitting of the 1000–1800 cm^−1^ spectral range with 5 bands. The red curve is the experimental spectrum of the AuNSs/ERGO layer; the black curve is the sum of the components D*, D, D″, G, and D′.

Raman spectra were studied to explore possible reasons for the beneficial effects of GO and ERGO on the electroreduction of H_2_O_2_. Typical results are presented in Figure 5. Two broad overlapping bands are observed, characteristic for the GO and related materials [33,34]. The relative band intensities and bandwidths vary, suggesting that the interaction with gold nanoparticles influences the GO structure. We analyzed all the spectra shown in Figure 5 by the 5-component fitting of the overlapping bands to five peaks: D, D′, G, D″, and D* (Gaussian/Lorentzian functions were used) and Figure 6 shows the results for AuNSs/ERGO as an example. The D and G bands correspond to the bands observed in graphene [35,36], and the relative intensity of the D band to the G band (*I*D/*I*G) is a popular measure of the number of defects in the graphene plane. López-Díaz et al. demonstrated the linear dependence between the ID/IG ratio and the diminishing percentage concentration of the sp^2^ carbons for a series of GO samples synthesized from different graphite materials (the percentage of sp^2^ carbons was obtained from the XPS data [34]). The D’ signal (ca. 1620 cm^−1^) is attributed to the disorder-induced phonon mode due to crystal defects [37]. The D” signal can be attributed to amorphous phases because its intensity diminishes as the crystallinity increases [34]. The position of the D” component is correlated to the oxygen content, showing the blue-shift with the increasing oxygen concentration [33]. Lee et al. showed that the D* (ca. 1150 cm^−1^) band intensity normalized to the G band increases with the increasing C/O atomic ratio [38]. This band is characteristic for the defects created due to the removal of oxygen atoms and signifies the presence of sp^3^ carbons. The presence of oxygen atoms also affects the G band position. It is blue-shifted with increased oxygen functional groups [39,40].

**Figure 7:**
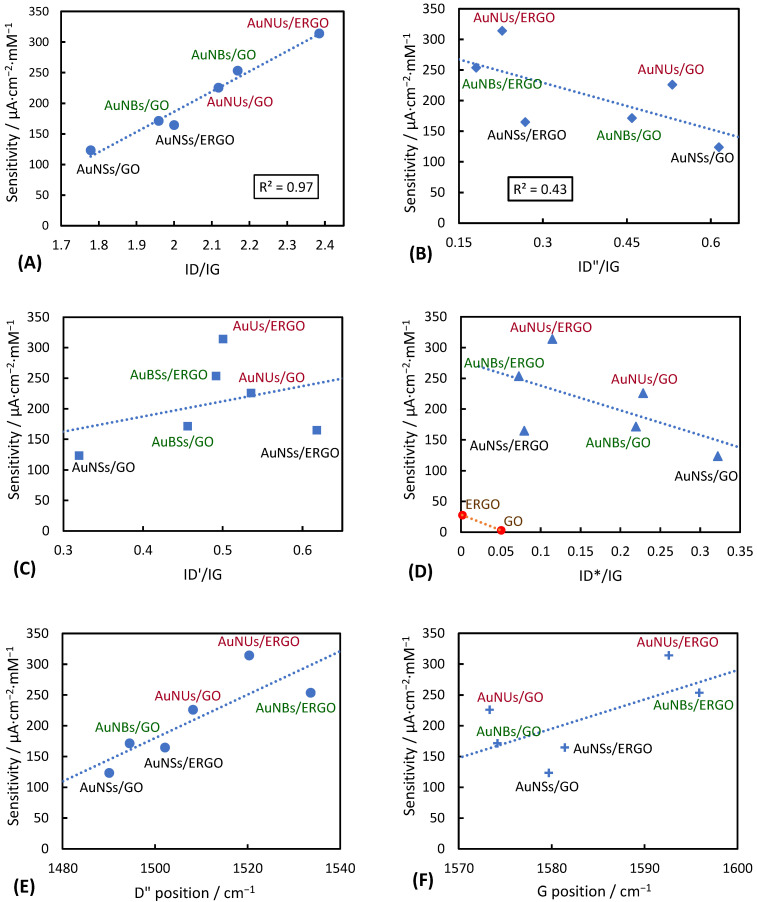
Sensitivities of the studied layers as the function of the parameters of Raman spectra: (**A**) as the function of the ID/IG ratio; (**B**) ID”/IG ratio; (**C**) ID’/IG ratio; (**D**) ID*/IG ration; (**E**) D” band position; (**F**) G band position.

The sensitivity values in Table 1 are plotted as the function of Raman spectral parameters like the positions of the spectral components obtained in the fitting procedure or the function of the component’s intensities normalized to the G band intensity (Figure 7). Among the spectral parameters studied, the ID/IG ratio shows the highest correlation with the sensitivity. The dependence is linear, with the R^2^ coefficient equal to 0.97. It indicates that the increasing number of defects benefits the sensitivity, and the decreasing percentage of sp^2^ carbons does not deteriorate the current intensities due to the H_2_O_2_ reduction. Another parameter describing defects in the GO samples is the ID”/IG ratio, indicating the presence of the amorphous phase [34]. The correlation between the ID”/IG value and the sensitivity is weaker. Still, the tendency is visible, and high sensitivity is obtained for the layers with low amorphous phase content. The crystallinity of the GO (or ERGO) layer is thus beneficial for the sensitivity.

The intensity of the D′ component, corresponding with crystallinity defects [33,34], is slightly higher for the most sensitive layers (Figure 7C), suggesting that such defects also contribute to the sensitivity to H_2_O_2_.

The ID*/IG value indicates the presence of the sp^3^ carbons in the GO structure. It is also sensitive to the carbon–oxygen atomic ratio, which is higher for the reduced GO samples compared to GO before reduction [38,41]. Lee et al. assigned D* to the motions of carbon atoms restricted by the oxygen-containing groups of GO [38]. We observe that the ID*/IG values are generally lower for the ERGO layers compared to GO layers for the same type of gold nanoparticles (Figure 7D). The same is observed for the ERGO and GO deposited directly on glassy carbon electrodes—without gold nanoparticles. Such unexpected changes in the ID*/IG value can be rationalized by the different compositions of the thermally and electrochemically reduced GO. Our previous studies [19] indicated that ERGO has only traces of -OH groups and no other oxygen-containing functionalities are present. The ERGO samples have no carbon atoms located by the oxygen-containing groups, and thus the intensity of D* is very low. Figure 7D shows that the sensitivity diminishes as the ID*/IG value increases, suggesting that sp^3^ carbon and sites with oxygen groups giving rise to the D* band are not beneficial for H_2_O_2_ electroreduction. The position of the D* is related to the oxygen content [33]. Our data show that it is weakly correlated with the sensitivity.

Other parameters correlated with the oxygen content are the position of the D” band and the position of the G band, which shift to a higher wavelength as the oxygen content increases. As shown in Figure 7E, the layers having high sensitivity are characterized by the D” band at a relatively high wavenumber. The same is valid for the G band (Figure 7F). As the positions of the D” and G band shift to high wavenumbers with the increasing oxygen content, Figure 7E,F suggest that the oxygen content is important for the electrocatalytic activity. However, the layers with ERGO are generally more sensitive than GO ones. This observation can be attributed to the possible higher electric conductivity of ERGO layers, a generally accepted fact. The electrochemical reduction of H_2_O_2_ probably needs a balance between the oxygen content, the conductivity, and the presence of defects (as indicated by Figure 7A).

## 3. Materials and Methods

### 3.1. Chemicals and Materials

Chemicals were commercial materials of the highest available grade, and they were used as received without any further purification. Sulfuric acid, tetrachloroauric acid (trihydrate, HAuCl_4_·3H_2_O, >99.9%), silver nitrate (AgNO_3_, 99.9999%), sodium citrate dihydrate, HOC(COONa)-(CH2COONa)_2_·2H_2_O (>99.9%), and sodium borohydride (powder, NaBH_4_, 98%) were purchased from Merck, Merck Life Science Sp.z.o.o, Poznan, Poland. Nitrogen gas (purity 99.999%) was purchased from Air Products (Warsaw, Poland). The 5 wt% Nafion solution was purchased from Merck Life Science Sp.z.o.o, Poznan, Poland. Hydrogen peroxide (H_2_O_2_, 30%) and potassium permanganate were purchased from Chempur (Piekary Slaskie, Poland). Phosphate buffer (pH = 6.5) was prepared using sodium dihydrogen phosphate (NaH_2_PO_4_) and disodium hydrogen phosphate (Na_2_HPO_4_) purchased from Avantor Performance Materials Poland S.A (Gliwice, Poland).

### 3.2. Synthesis of Gold Nanoparticles

The fabrication of AuNSs, AuNUs, and AuNBs was carried out according to the procedures described in our previous publications [42,43]. The resulting colloidal suspensions containing AuNSs, AuNUs, and AuNBs were centrifuged, and the supernatant solutions were removed and subsequently replaced with water. The final centrifuged solutions of the water-dispersed AuNSs, AuNUs, and AuNBs were 20 times more concentrated. As a rule, appropriate amounts of the resulting water-dispersed AuNSs, AuNUs, and AuNBs were dropped onto the surfaces of the glassy carbon electrode to obtain loadings of gold nanostructures equal to 60 µg cm^−2^ and left to dry. Then, the electrodes were covered by 1 µL of Nafion–ethanol mixture (1:10) and left to dry.

### 3.3. Synthesis of GO

Graphene oxide (GO) was synthesized from the graphite powder (Sigma-Aldrich, St. Louis, MO, USA; 1–2 µm, synthetic) using the modified Hummers–Offeman method [44]. In brief, 6 g of the graphite powder was added to 150 mL of concentrated sulfuric acid. The ingredients were mixed while being cooled in an ice bath to avoid overheating. Subsequently, 21 g of potassium permanganate was added under stirring (slowly, to keep the temperature below 30 °C). After 2 h of stirring, 150 mL of distilled water was added slowly, and then 35 mL of hydrogen peroxide was added. The as-prepared GO was centrifuged at 6000 rpm for 30 min, washed four times with distilled water, and left to dry. GO obtained with this procedure shows a high epoxide group content (as controlled by the FT-IR spectra 1225 cm^−1^ band).

### 3.4. Preparation of the Electrodes

The GC electrodes modified with the AuNSs, AuNUs and AuNBs were conditioned via repetitive potential cycling between −0.5 and +0.3 vs. SCE in a de-aerated phosphate buffer solution (pH = 6.5) at a scan rate of 50 mV/s until a stable response was obtained. As-prepared electrodes were studied either without further modification, or they were modified by GO.

To obtain electrodes modified by AuNUs/GO, AuNBs/GO, and AuNSs/GO, the 30 μL of GO solution (7.5 mg/mL, in ethanol) was drop-casted onto electrodes covered by AuNUs, AuNBs, and AuNSs and left to dry at room temperature. The preparation of electrodes covered by AuNUs/ERGO, AuNBs/ERGO, and AuNSs/ERGO involved an additional step of reducing GO according to the voltamperometry technique. The electroreduction of GO was carried out by applying five voltammetric cycles between −0.4 V and −1.2 V vs. SCE with a scan rate of 5 mV/s in de-aerated phosphate buffer solution (pH = 6.5).

### 3.5. Electrochemical Setup

All electrochemical experiments were performed in a conventional three-electrode cell using PGSTAT 204 (Metrohm Autolab, Utrecht, The Netherlands). A saturated calomel electrode (SCE) was the reference electrode—ca. 240 mV relative to the reversible hydrogen electrode (RHE). The counter electrode was a carbon rod.

The rotating disk electrode from Pine Instruments was used to mix the solution after H_2_O_2_ additions during amperometric experiments.

Before each experiment, the working electrode was polished on a wet Buehler polishing cloth containing alumina slurry (5 μm grain size) until it was mirror-like. After polishing, it was kept for ca. 5 min in an ultrasonic bath in distilled water to remove the alumina traces and rinsed few times with fresh distilled water.

### 3.6. Raman Spectra

The Raman experiments were carried out using a DXR Raman spectrometer (Thermo Fisher Scientific, Waltham, MA, USA). The instrument was operated using the 633 nm excitation line and the 600 lines/mm diffraction grating. The Olympus M Plan N, 50×/0.75, ∞/0/FN 22 objective was used. The signal was collected from the ca. 1 µm^3^ spot in the configuration used. The laser beam was focused on top of the layer to minimize the contribution of the GC support to the spectra. Typically, 2 mW laser power was used. The spectra were averaged over 25 random points on the sample to ensure reproducibility.

The deconvolution of spectral bands was carried out using the OMNIC program (Thermo Scientific).

### 3.7. Transmissive Electron Microscopy

The nanoparticles were studied by HRTEM using a Talos F200X, FEI instrument (Thermo Fisher Scientific, Waltham, MA, USA). The applied voltage was equal to 200 kV.

## 4. Conclusions

We demonstrate that amperometric signals due to the H_2_O_2_ reduction depend on the shape of nanoparticles. The order of sensitivities is the following: AuNSs < AuNBs < AuNUs. We attribute this order to the increasing surface area of the nanoparticles and presence of edges in AuNBs and AuNUs. Covering the layer of the gold nanoparticles by the GO or ERGO layer results in synergetic electrocatalytic current enhancement. The effect is largest for the AuNUs/ERGO system.

Raman spectra indicate that the interaction between gold nanoparticles and GO (or ERGO) affects the GO structure. The correlation of the Raman spectra of studied layers with the sensitivities to H_2_O_2_ shows that the GO or ERGO layer defects are pivotally important for high current signals. The correlation between the ID/IG intensities ratio and the sensitivity is linear, with an R^2^ factor equal to 0.97. Other spectral parameters indicate that the oxygen content has a relatively low impact on sensitivity.

## Figures and Tables

**Table 1:** The sensitivities of the studied electrodes.

Electrode	Sensitivity [µA·cm^−2^·mM^−1^]	Limit of Detection * [µM]
GO	1.4	83.3
ERGO	27.7	50.1
AuNSs	112.6	18.2
AuNSs/GO	123.5	21.1
AuNSs/ERGO	164.7	24.0
AuNBs	151.5	12.8
AuNBs/GO	171.4	21.2
AuNBs/ERGO	253.5	21.1
AuNUs	202.2	11.0
AuNUs/GO	225.8	18.1
AuNUs/ERGO	314.1	8.5

* Limit of detection was calculated as 3·noise/sensitivity.

**Table 2:** The real surface areas of studied electrodes.

Electrode	Real Surface Area [cm^−2^]
GO	0.311
ERGO	0.633
AuNSs	0.498
AuNSs/GO	0.335
AuNSs/ERGO	0.566
AuNBs	0.569
AuNBs/GO	0.211
AuNBs/ERGO	0.615
AuNUs	0.818
AuNUs/GO	0.611
AuNUs/ERGO	0.896

## Data Availability

Data are contained within the article.
